# Lessons learned from continued TB outbreaks in a high school

**DOI:** 10.1371/journal.pone.0188076

**Published:** 2017-11-16

**Authors:** Young Kim, Byeong Ki Kim, Hong Jo Choi, Sung Weon Ryu, Eui Sook Kim, Yoon Soo Chang, Hee Jin Kim, Jae Hyung Cha, Je Hyeong Kim, Chol Shin, Seung Heon Lee

**Affiliations:** 1 Division of Pulmonary, Sleep and Critical Care Medicine, Department of Internal Medicine, Korea University Ansan Hospital, Korea University College of Medicine, Ansan, Republic of Korea; 2 Yonsei University College of Medicine, Seoul, Republic of Korea; 3 Korea Institute of Tuberculosis, Osong, Republic of Korea; 4 Public Healthcare Center, Ansan, Republic of Korea; 5 Medical Science Research Center, Korea University Ansan Hospital, Korea University College of Medicine, Ansan, Republic of Korea; Indian Institute of Technology Delhi, INDIA

## Abstract

We investigated the aftereffects of confirmatory QuantiFERON testing (QFT) added to a positive tuberculin skin test (TST). We reviewed the pre and post course of sequential tuberculosis (TB) outbreaks in a high school where massive 43 active TB cases had been found within one year before delayed contact investigation. And we investigated the TB development in relation to initial TST and QFT during mean follow-up of 3.9 ± 0.9 years. After delayed contact investigation for two subsequent TB outbreaks, 925 contacts were divided into the following 3 groups: TST- (n = 632), TST+/QFT+ (n = 24), TST+/QFT- (n = 258). QFT- was more prevalent than QFT+ in contacts with 10mm ≤ TST <15mm (158, 61.2%) compared with TST ≥15mm (100, 38.8%) among the TST+ reactors (P < 0.001). Among the 258 TST+/QFT- subjects, 256 received no latent TB infection (LTBI) treatment, but 7 contacts developed TB during follow-up. Among these 7 patients, 4 had initial TST ≥15mm and 3 had 10mm ≤ TST <15mm. In conclusion, the delayed contact investigation for LTBI in a high school resulted in continued TB developments. False-negative QFT performed late among the TST+ reactors should not be considered criteria for LTBI treatment. Additionally, the contacts only with TST ≥15mm should be considered for LTBI treatment in congregate settings of intermediate-burden countries.

## Introduction

To eliminate tuberculosis (TB), rapid diagnosis and treatment of infectious TB patients in TB high-burden countries and control of latent TB infection (LTBI) for TB contacts in low-burden countries are important main strategies [1,2]. However, how to control LTBI in intermediate-burden countries such as South Korea where the incidence rate of active TB is 80/100,000 [3] is not well understood, although WHO recommends the tuberculin skin test (TST) and interferon-gamma release assay (IGRA) guidelines based on TB burden with a cut-off level of 100/100,000 that discriminates the high from low burden in addition to economic status [4,5].

Traditionally, the TST has been used to identify LTBI preferentially in children and adolescents. The WHO recommends that IGRA should not replace TST in low-income countries, but can replace the TST in high-income and upper middle-income countries with estimated TB incidence less than 100/100,000 [5]. Recently, the United States recommends the IGRA single test can be used for the diagnosis of latent TB in all cases except for subjects less than 5 years of age [6] and the United Kingdom recommends the IGRA can be offered as an additional test for individuals 2–17 years of age with an initial negative TST result [7]. However, in several countries, a two-step strategy consisting of an initial TST and confirmatory IGRA was utilized which was useful in high-income countries [8]. The Korean LTBI policy, which adopted a two-step strategy for contact investigation during school outbreaks has been expanded with few accumulated evidences following the changing trend in world LTBI guidelines.

Even though IGRA have many merits compared with TST in the aspect of convenience and interpreters’ errors except high costs, other disadvantages of using confirmatory IGRA added to a positive TST should be considered during public contact investigations. Missed identification of true LTBI due to false-negative IGRA in TB outbreaks of schools or military communities consisting of 2–3 years of community life can result in continued TB outbreak situations accompanied by socioeconomic loss [8]. When using a two-step screening strategy, performing an IGRA test within 3 days of administering the TST is safe to prevent spontaneous conversion and reversion [9]. In addition, variability of Quanti-FERON-TB Gold In-Tube test (QFT; Cellestis Ltd, Carnegie, VIC, Australia) results can occur due to technical errors caused by impact of blood volume and tube shaking, therefore these errors should be avoided [10].

In this study, we identified the disadvantages of the two-step strategy (a TST followed by an IGRA for TST+ reactors) and the meaning of strong positive TST results after a TB outbreak in congregate settings such as high schools.

## Methods

### Change in Korean TB control policy for contact investigations

The contact investigation policy for TB outbreaks was reinforced after 2011 when the standardized protocol for contact investigation was implemented [11]. In 2013, a systematized contact investigation team from Korean Centers for Disease Control and Prevention (KCDC) was launched. Before the update of the 2017 Korea LTBI guideline recommending a TST or IGRA as a diagnosis tool for TB close contacts 5–18 years of age, a TST followed by an IGRA (the two-step strategy) for contact investigation of a TB outbreak was the conditionally adopted strategy.

### TB outbreaks and subsequent approach

In 2007, multiple TB outbreaks in middle and high schools occurred in the local city of South Korea where one tenth of 700,000 residents were immigrants and the TB incidence rate (314/100,000) was significantly higher than the average rate for South Korea (97/100,000) during the same period [12].

Initially, a TB outbreak in a high school was reported to the public health care center. During an epidemiologic investigation, the isolated strains from this TB outbreak were the same strains based on restriction fragment length polymorphism (RFLP). All close contacts from the TB outbreaks were adolescents in the same school; all 947 students were analyzed for this retrospective review. Using the data from the 2005–2007 TB outbreaks, we reviewed the TB outbreak courses of a high school and identified the TB development associated with the initial TST and QFT results using the Korean national claims database.

### TST and QFT

The TST was administered by intradermal injection (0.1 ml) of2 tuberculin units of purified protein derivative (RT 23; Statens Serum Institute, Copenhagen, Denmark) into the anterior surface of the forearm with a disposable syringe and a 27-gauge needle by using the Mantoux technique. Induration was measured after 48–72 hours with a ruler or a caliper by an expert nurse. The QuantiFERON TB Gold In Tube test (QFT; Cellestis Ltd, Carnegie, VIC, Australia) was performed as a second-step test among TST+ (cut-off ≥10mm) reactors, and interpreted according to the manufacturer’s instructions. Whole blood was collected by venipuncture from each subject and incubated for 16–24 hours in 3 separate conditions. A QuantiFERON value of 0.35 international units or more was deemed positive. Contact investigation with LTBI treatment policies in TB outbreak are mandatory under the Korean national tuberculosis control program (NTP) since 2007 [11]. Therefore, the requirement for informed consent was waived and this study was approved by the Institutional Review Board of the Korea University Ansan Hospital, Ansan, South Korea.

### Statistical analysis

All analyses were performed using SPSS software, version 20.0 (SPSS Inc., Chicago, IL, USA). The χ2 tests were used to determine the differences between groups based on the TST cut-off value. Logistic regressions using different variables were done for the prediction of TB development. And, ROC (Receiver Operating Characteristic) curves were constructed to establish the optimal cut-off points of TST induration size. All tests for significance were 2-tailed and P-values < 0.05 were considered statistically significant.

## Results

### Continued TB outbreaks without LTBI diagnosis and treatment

**Fig 1:**
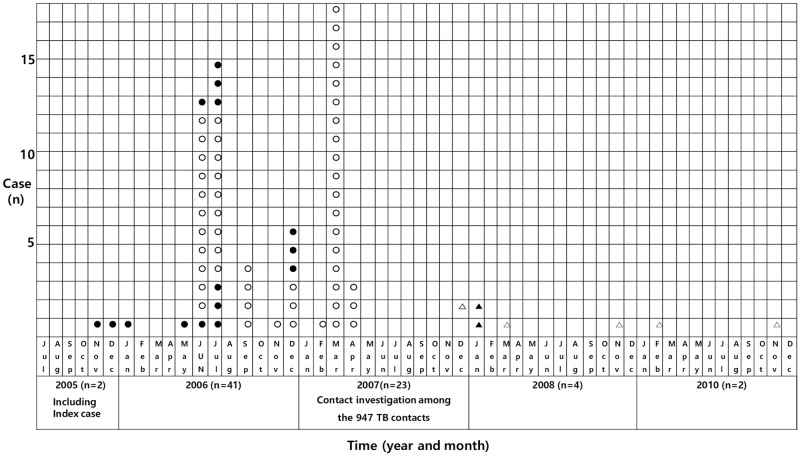
Scheme of TB outbreak in a single high school in Korea. Each symbol in column represents one student and placed at the point at which the student was diagnosed as active TB.**●** = Active TB cases with positive AFB culture; **○** = Clinical TB cases with negative AFB culture; ▲ = Active TB cases with positive AFB culture and smear developed during follow-up among contacts who were TST+/QFT-; **Δ** = Clinical TB cases with negative AFB culture developed during follow-up among contacts who were TST+/QFT-; TB = tuberculosis; AFB = acid fast bacilli; TST = tuberculin skin test; QFT = QuantiFERON-TB Gold In-Tube test.

**Fig 2:**
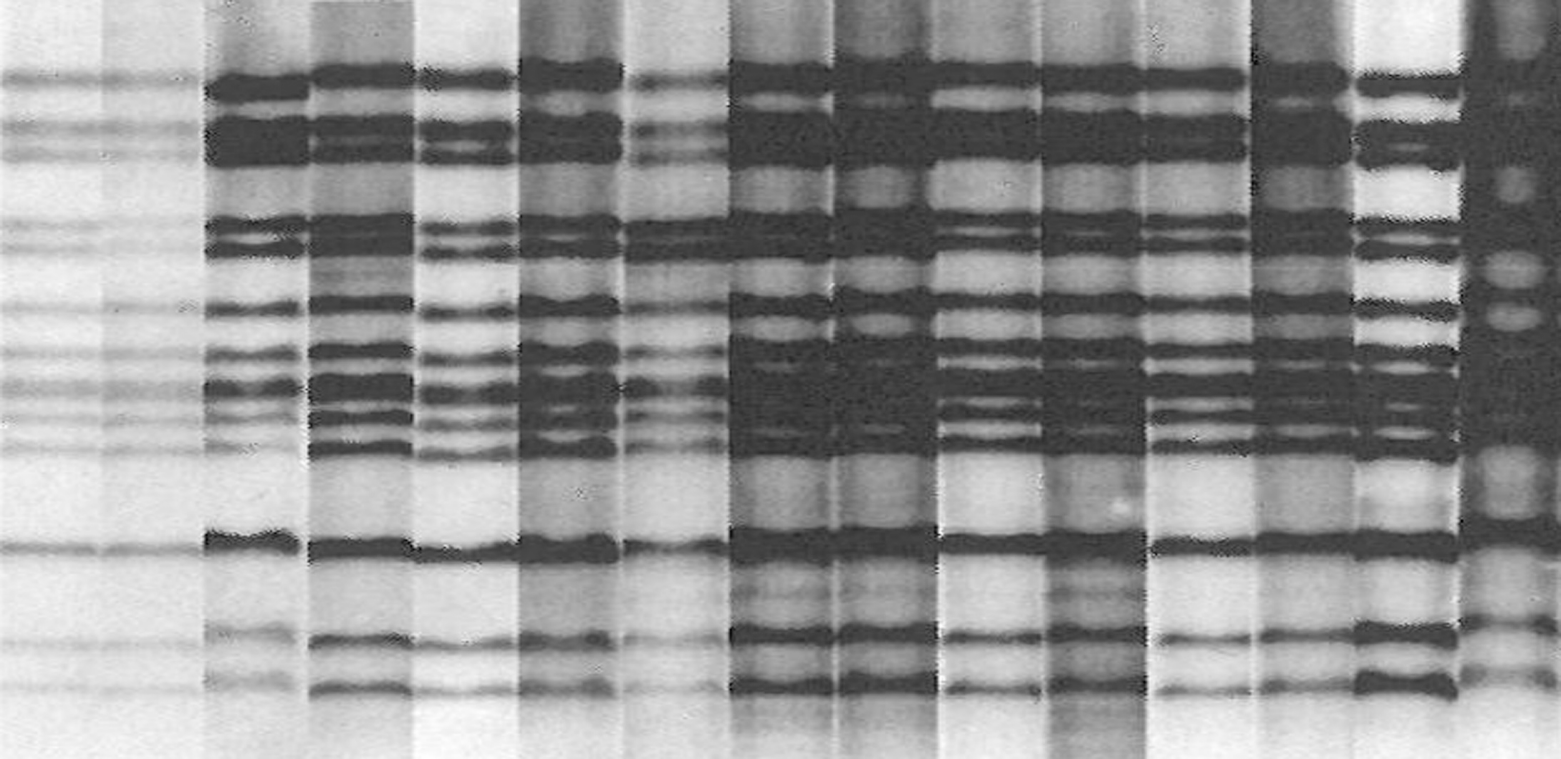
RFLP results of 15 active TB cases with positive AFB culture. The banding patterns of 15 outbreak strain isolates are completely same. RFLP = restriction fragment length polymorphism; TB = tuberculosis; AFB = acid fast bacilli.

From November 2005, when infectious source cases of TB developed, a total of 3 active TB cases developed until January 2006 in a high school. Four months after this event, 12 more sequential active TB cases with positive AFB culture were identified and 28 more clinical TB cases with abnormal chest X-rays (CXRs) were diagnosed from May 2006 to December 2006 based on the results from mass screening with CXR and sputum AFB smears and cultures (Fig 1). After RFLP fingerprinting, a total of 15 active TB cases with positive AFB cultures were identified as the same strain (Fig 2). Belated contact investigation with TST and QFT was conducted in January 2007.

### Clinical characteristics of TB contacts

We analyzed 947 contacts (468, 49% males; 479, 51% females) among high school students. The clinical characteristics of the subjects are presented in Table 1. The mean age of the contacts was 17.5 years (range 17–18 years). Among the 947 contacts, 21 (2.2%) had a family history of TB; 1 (0.1%) had a history of previous anti-TB treatment; 616 (65.0%) had BCG scars; TST induration size was ≥10mm in 282 (29.8%) and >15mm in 122 (12.9%) (Table 1).

**Table 1:** Clinical characteristics of TB contacts.

Clinical characteristics	Values (N = 947)
Gender (Male:Female)	468 (49%):479 (51%)
Mean age (years)	17.5 (range 17–19)
History of previous anti-TB treatment, %	1 (0.1%)
Family history of TB, %	21 (2.2%)
Presence of a BCG scar, %	616 (65%)
TB diagnosed with screening CXR and chest CT, %	22 (2.3%)
TST+ (≥10mm) among the contacts, %	282(29.8%)
TST ≥15mm among the contacts, %	122 (12.9%)
QFT+* among subjects with TST+, %	24 (8.5%)
10mm ≤ TST <15mm	2 (8.3%)
TST ≥15mm	22 (91.7%)
QFT-* among subjects with TST+, %	258 (91.5%)
10mm ≤ TST <15mm	158 (61.2%)
TST ≥15mm	100 (38.8%)
Mean duration of follow-up, years	3.9; SD ± 0.9

TB = tuberculosis; BCG = bacillus Calmette-Guérin; CXR = Chest X-ray; TST = tuberculin skin test; QFT = QuantiFERON-TB Gold In-Tube test; SD = standard deviation.

*TST first, followed by QFT when TST was positive.

### Results of contact investigation

**Fig 3:**
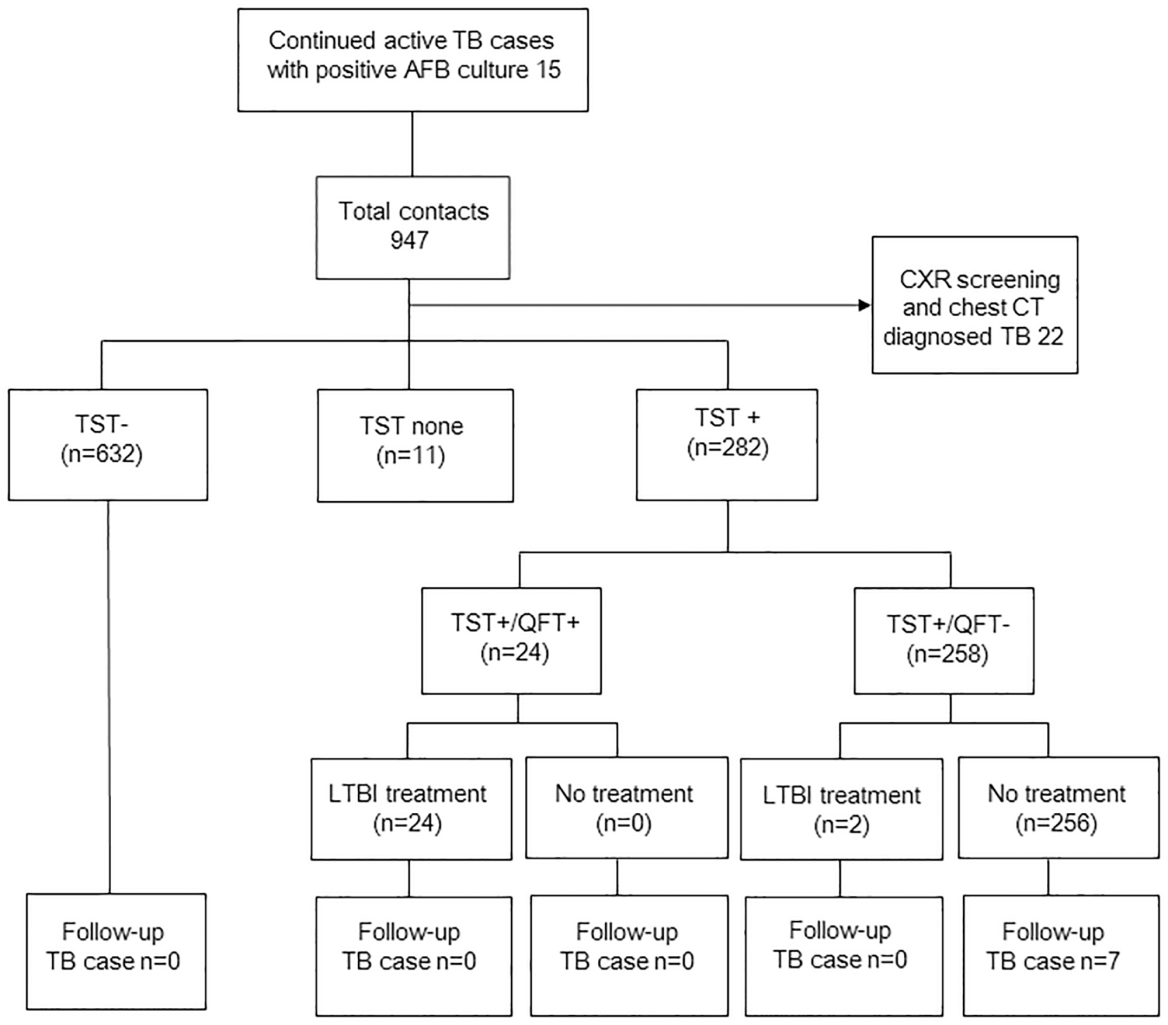
Flow diagram for TB contacts of infectious patients with TB. All 947 contacts excluding 22 active TB cases were divided into separate groups based on the TST and QFT results. The TB developments of contacts were traced during the mean duration of 3.9 years TB = tuberculosis; TST = tuberculin skin test; QFT = QuantiFERON-TB Gold In-Tube test; LTBI = latent TB infection.

Initial TB was diagnosed with CXR and chest CT in 22 subjects (2.3%). The baseline CXRs of 925 contacts excluding active TB cases were normal. The TST and QFT results of the 925 TB contacts were as follows: TST- (n = 632), TST none (n = 11), TST+ (n = 282), TST+/QFT+ (n = 24) and TST+/QFT- (n = 258) (Fig 3). The second-step QFT was conducted in all 282 TST+ subjects. Among the TST+ reactors (282/925 contacts, 30.5%), a second-step QFT showed positive results in 24/282 (8.5%). Among the 24 TST+/QFT+ subjects, 22 had TST ≥15mm (76.0%) and 2 had 10mm ≤ TST <15mm (24.0%); All 24 TST+/QFT+ subjects received LTBI treatment. The second-step QFT showed negative results in 258/282 (91.5%). Among the 258 TST+/QFT- subjects, 100 had TST ≥15mm (38.8%) and 158 had 10mm ≤ TST <15mm (61.2%); 256 received no LTBI treatment and 2 subjects received treatment (Fig 3).

When the results of QFT tests among the TST+ reactors based on the TST cut-off value (10mm) were analyzed, QFT- was more prevalent than QFT+ in contacts with 10mm ≤ TST <15mm (158, 61.2%) compared with TST ≥15mm (100, 38.8%; odds ratio 17.38, C.I:4.0–75.5, P < 0.001; Table 2).

**Table 2:** The results of QFT tests among the TST+ reactors based on TST cut-off value.

	QFT- (N = 258)	QFT+ (N = 24)	P-value	Odds ratio
10mm ≤TST <15mm	158 (61.2%)	2 (8.3%)	P < 0.001	17.380 (4.0–75.5)
TST ≥15mm	100 (38.8%)	22 (91.7%)		Ref.

QFT = QuantiFERON-TB Gold In-Tube test; TST = tuberculin skin test; Ref. = reference.

### TB among the TST+/QFT- contacts

Using the database, we investigated the progression of TB among TB contacts within a 3-year follow-up period after TST. Seven contacts developed TB during the follow-up (Fig 3, Table 3). All were TST+/QFT- and received no LTBI treatment. One patient was sputum smear-positive for TB with cavitary lung lesions on CXR and previous TST size of 15mm. One patient was sputum culture-positive for TB with cavitary lung lesions on CXR and previous TST size of 15mm. Four patients had culture-negative clinical TB with cavitary lung lesions on CXR and previous TST sizes of 18mm, 15mm, 11mm and 13mm. One patient had culture-negative clinical TB with pleurisy on CXR and a previous TST size of 10mm. In total, 4 patients had previous strong positive TST results ≥15mm (Table 3). Among subjects with TST induration ≥15mm and QFT- contacts, the rate of TB progression was 4/100 (4.0%) and among subjects with TST induration size ≥10mm and <15mm, the rate of TB progression was 3/158 (1.9%).

**Table 3:** Clinical characteristics of 7 contacts who developed TB during follow-up.

Patient	Age at TST	Sex	TST date	TST size	QFT result	LTBI treatment	TB diagnosis date	Sputum AFB	CXR
1	17	M	2007. 1.23,	18mm	Neg.	No	2010. 11.1	Neg.	Cavity
2	18	M	2007. 1.22	15mm	Neg.	No	2008. 1.8	Pos.	Cavity
3	18	M	2007. 1.22	15mm	Neg.	No	2008. 1.3	Pos.	Cavity
4	18	M	2007. 1.22	15mm	Neg.	No	2008. 11.17	Neg.	Cavity
5	18	M	2007. 1.22	10mm	Neg.	No	2008. 3.4	Neg.	Cavity with Pleurisy
6	18	M	2007. 1.22	11mm	Neg.	No	2010. 2.1	Neg.	Cavity
7	18	F	2007. 1.22	13mm	Neg.	No	2007. 12.27	Neg.	Cavity

TST = tuberculin skin test; QFT = QuantiFERON-TB Gold In-Tube test; LTBI = latent TB infection; AFB = acid fast bacilli; CXR = chest X-ray; Neg. = negative; Pos. = positive.

**Fig 4:**
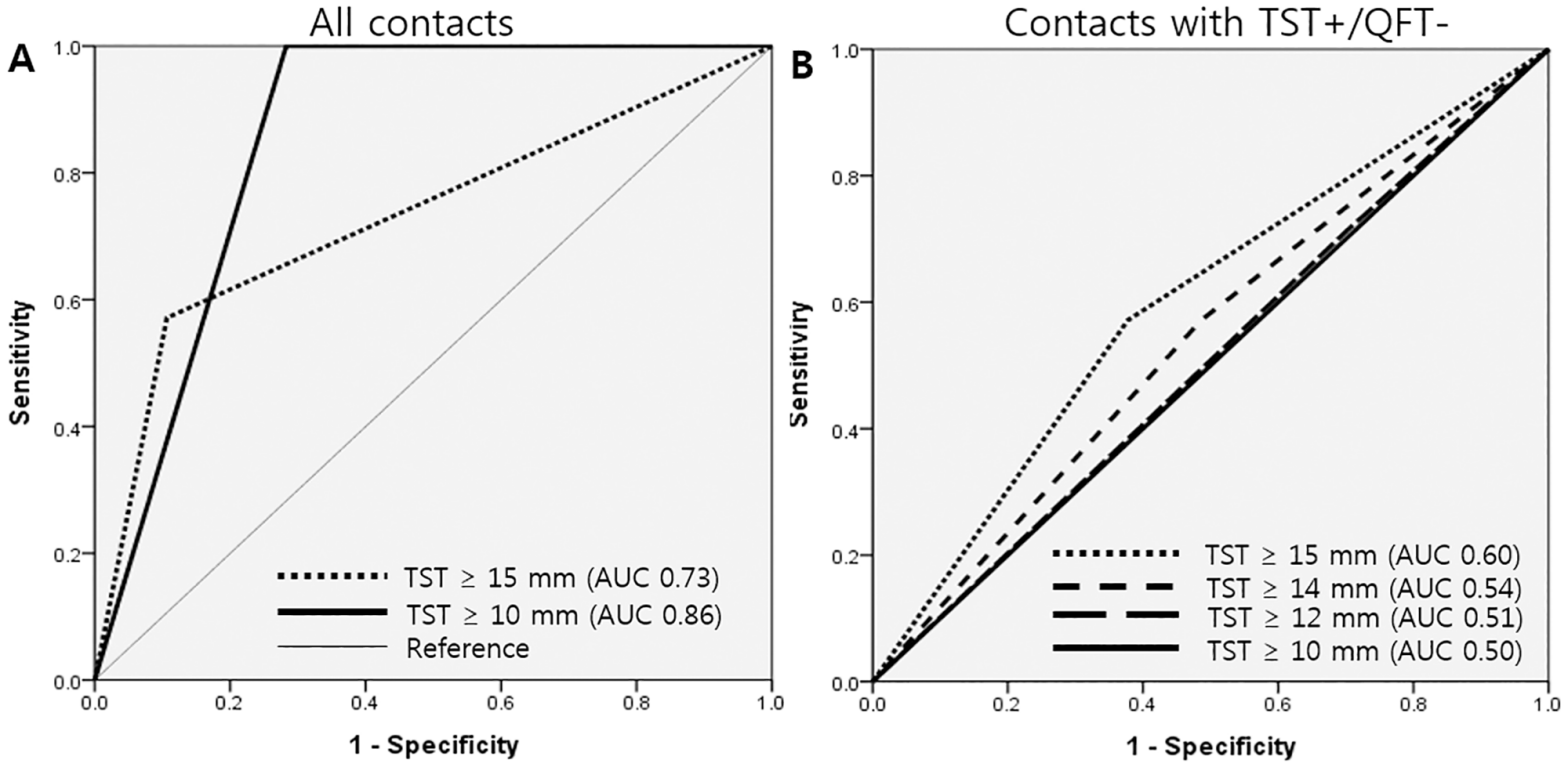
Receiver Operating Characteristic (ROC) curves for prediction of TB cases using different cut-off points of TST induration size. (A) For all contacts, the overall accuracy was higher with the criteria of TST ≥10 mm than with that of TST ≥15 mm to predict TB cases. (B) But, for the contacts with TST+/QFT- results, the overall accuracy was the most highest with the criteria of TST ≥15 mm. TST = tuberculin skin test; AUC = Area under the curve.

The ROC (Receiver Operating Characteristic) curve was constructed to establish the optimal cut-off points of TST induration size after exclusion of the contacts who received LTBI treatment. ROC curve for the prediction of TB cases in this study showed that the criteria of TST ≥10 mm (AUC 0.86) was more accurate than that of TST ≥15 mm (AUC 0.73) to predict TB cases for all contacts. However, for the contacts with TST+/QFT- results, the overall accuracy was the most highest with the criteria of TST ≥15 mm (Fig 4).

## Discussion

The delayed contact investigation for LTBI in a high school TB outbreak caused continuous successive TB outbreaks and the presumed false-negative results of QFT conducted among the TST+ reactors led to confusing decision with no LTBI treatment based on the two-step strategy in LTBI policy.

This disaster which represented continued TB outbreaks in a high school occurred in South Korea, a country with intermediate TB burden before the intensified policy for LTBI diagnosis and treatment in 2011 showed the contagious TB spread in condensed environments such as a high school. All 7 students with TST+/QFT- results who progressed to TB with cavity on CXR after 1 year of contact investigation in January 2007 had been first and second grade students in the same high school and 4 had showed TST induration size ≥15mm. In particular, 5 patients in the same grade including 2 patients with positive sputum AFB smear and positive sputum AFB culture developed TB from December 2007 to 2008. Also this second outbreak of TB caused a burden of repeated contact investigation in 2008. Therefore, lower grade students with a strong induration size of TST (≥15mm) should be aggressively treated for LTBI considering the remaining long period of high school attendance even without a confirmatory QFT test after TB contact investigation.

The IGRA test including QFT are dynamic assays where interferon gamma values can fluctuate around the cut-off value, leading to conversion or reversions [13], although the immune status of the host [14] or completion of LTBI treatment can affect the IGRA dynamicity [15]. In the same context, low positive rate of QFT (8.5%, 24/282) among the TST positive reactors and the false-negative QFT results even in the strong positive TST (≥15mm) reactors apparently seem to originate from spontaneous reversion of the QFT test rather than technical error [16,17] because QFT tests were delayed by more than 3 months after the initial TB outbreak; Nevertheless our results showed a strong positive TST (TST ≥15mm) tended to be associated with QFT+ compared with weak positive TST (10mm ≤ TST < 15mm) (Table 2) [15].

Although the initiation of the infectious period cannot be determined, an assigned initiation of 3 months before a TB diagnosis is recommended based on the TB patient’s index characteristics such as cavity on CXR [18]. Therefore, QFTs conducted for a retracement of contacts after an active TB should not be preferred to TST due to possible reversion of the QFT [18].

Recently in the United States, the 2016 American Thoracic Society, Centers for Disease Control and Prevention, and Infectious Diseases Society of America recommends the use of IGRA or TST for the diagnosis of latent TB in all cases, but recommends IGRA for subjects over 5 years of age [6]. In the United Kingdom, the NICE guideline have been recently updated and recommends using IGRA alone for subjects 18–65 years of age if a large number of people require screening. In particular, for individuals 2–17 years of age, LTBI should be screened only using TST for contacts with infectious TB patients and when the initial TST is negative, additional IGRA tests should be performed after 6 weeks and the TST repeated [7]. In the same context, based on the results from our report, the two-step strategy should be modified for Korean children and adolescents using additional evidence from large scale studies in South Korea.

The present study had several limitations. First, we could not determine if the TB strains from the 7 contacts that developed TB during follow-up were the same as the TB strain from the index case. Second, in this study, QFT was significantly delayed after first TB outbreak and the findings were limited to only a single school, therefore, the findings cannot be generalized. To propose a basis for amending Korean guidelines, the confirmatory IGRA test for the TST positive reactors should be reverified based on concrete evidence from studies where QFT is performed on a large number of subjects.

In conclusion, false-negative QFT among TST+ reactors could cause continuous sequential TB outbreaks with the use of confirmatory IGRA tests among the TST+ reactors (two-step strategy) for LTBI treatment in school TB outbreaks. Furthermore, the contacts with a strong induration size of TST (≥15mm) should be considered for LTBI treatment without a confirmatory IGRA test especially in intermediate-burden countries.
